# Anti-Desmocollin Autoantibodies in Autoimmune Blistering Diseases

**DOI:** 10.3389/fimmu.2021.740820

**Published:** 2021-09-10

**Authors:** Xavier Bosch-Amate, Pilar Iranzo, Marta Ivars, José Manuel Mascaró Galy, Agustín España

**Affiliations:** ^1^Dermatology Department, Hospital Clínic de Barcelona, Universitat de Barcelona, Barcelona, Spain; ^2^Dermatology Department, University Clinic of Navarra, University of Navarra, Madrid, Spain; ^3^Dermatology Department, University Clinic of Navarra, University of Navarra, Pamplona, Spain

**Keywords:** autoimmune blistering disease, pemphigus, desmocollin, desmoglein, systematic review, antibody

## Abstract

The presence of anti-desmocollin (Dsc) antibodies is rarely described in autoimmune blistering diseases patients. Moreover, several clinical phenotypes of pemphigus may be associated with these antibodies. In this review we analyze clinicopathological, immunologic and outcome features of anti-Dsc autoimmune blistering diseases patients, to improve their diagnosis and management. We conducted a systematic search of PubMed and Embase (1990-present) for studies reporting cases of autoimmune blistering diseases with anti-Dsc antibodies. We classified the selected patients as patients with exclusively anti-Dsc autoantibodies, and patients with anti-Dsc and other autoantibodies. Of 93 cases with anti-Dsc autoantibodies included, 38 (41%) had exclusively these antibodies. Only 18% of patients presented with the typical clinicopathological phenotype of pemphigus vulgaris or pemphigus foliaceous. Mucosal involvement was seen in approximately half of the patients. Up to 18% of cases were associated with neoplasms. Acantholysis was described in 54% of cases with histopathological information. Treatments and outcomes vary in the different clinical phenotypes. The presence of anti-Dsc antibodies must be suspected mainly in those patients with either atypical pemphigus, in special with clinical pustules, or in cases showing intraepithelial or dermal neutrophilic/eosinophilic infiltrate on histological examination and dual pattern by direct immunofluorescence examination.

## Introduction

Autoimmune blistering diseases (AIBD) encompass a group of disorders characterized clinically by the development of cutaneous and/or mucosal blisters and erosions. Patients synthesize autoantibodies against structural proteins of epidermis and/or dermal-epidermal junction. Pemphigus is a AIBD skin disease caused by the production of autoantibodies that target desmosomal adhesion molecules including desmoglein (Dsg) and desmocollin (Dsc), among others (1). Pemphigus diagnosis is based on clinical, histological, direct immunofluorescence (DIF) and serological findings (2). Dsg3 and Dsg1 are the major target antigens in pemphigus, but in the last 20 years, new relevant findings have demonstrated the role of non-Dsg autoantibodies in pemphigus cell-cell detachment (3, 4) even acting synergistically when Dsg autoantibodies are absent (5). Dsc are non-Dsg cadherin family members with three different isoforms, Dsc1, 2, and 3. Dsc1 and 3 were among the top 10 most commonly recognized antigens in pemphigus vulgaris (PV) patients sera (6). Anti-Dsc antibodies have mostly been involved in atypical pemphigus such as pemphigus herpetiformis (PH) and pemphigus vegetans (PVeg) (7).

The primary objective of this study was to review the clinical, histologic and immunologic features of AIBD, mainly pemphigus, with anti-Dsc antibodies in order to better diagnose these patients. The secondary objective was to review treatment responses and suggest some clues to the management of these disorders.

## Methods

### Literature Review and Article Selection

A literature review of PubMed and Embase (1990-present) databases of AIBD cases with anti-Dsc autoantibodies was conducted using the terms: desmocollin, non-desmoglein autoantibodies, autoimmune blistering disease, pemphigus, atypical pemphigus, pemphigus herpetiformis, IgA pemphigus, IgG/IgA pemphigus, paraneoplastic pemphigus (PNP) and pemphigus vegetans. Articles published in English, or those written in other languages with an English abstract were considered for eligibility. Only human patients were included, and purely serological studies without clinical information were excluded. Articles whether online, in print, or in press from all years were included in the analysis. Reference lists of included articles in primary search were further screened for additional eligible publications. Three representative cases have also been included in the analysis and are shown as Figures.

### Data Extraction

Each relevant article was critically reviewed. The variables analyzed were: demographics, clinical manifestations, histopathologic and immunopathologic findings, immunological profile, treatments and their outcomes, and associated comorbidities. We considered all patients with annular plaques with or without vesicles/pustules/blisters as PH, and IgA pemphigus was considered as subcorneal pustular dermatosis (SPD) or intraepidermal neutrophilic dermatosis (IEND) based on the clinicopathological description from the author. When available, histologic images were reviewed to complete original authors’ text descriptions. In patients with a shift in the clinical or immunological features during the disease course, we included the latest form. We classified the selected patients as: 1) Patients with exclusively anti-Dsc autoantibodies, and 2) Patients with anti-Dsc and other autoantibodies.

## Results

Various techniques were used to detect anti-Dsc autoantibodies: Immunoblot, immunoprecipitation, indirect immunofluorescence (IIF) with COS7 cells transfected with Dsc and ELISA with baculovirus-produced recombinant proteins or expressing human recombinant proteins in mammalian cells. Most of the described cases were Japanese or written by Japanese authors, since anti-Dsc antibodies have mainly been detected in Japanese labs. A total of 93 cases with anti-Dsc autoantibodies were analysed. Of these, 38 patients had exclusively anti-Dsc autoantibodies. Overall, there were no significant gender differences and the presentation age was variable, with predominance in middle age. Eleven patients (12%) had a clinicopathological phenotype of PV and only 6 (6%) had a PF phenotype.

### Patients With Exclusively Anti-Desmocollin Autoantibodies

#### Patients With Exclusively IgG Anti-Desmocollin Autoantibodies

Fourteen papers including 17 patients were selected (**Supplementary Table 1**) (8–21). There was no sex predominance and age varied between 11 and 83 years (median 64,5). PH and PVeg were the most common phenotypes (9 and 5 patients, respectively). Mucosal involvement was found in 6 patients (8, 10, 16, 18, 21), and was the only manifestation in 2 of them (8, 16). Acantholysis was present in 11 biopsies, with a predominant eosinophilic infiltrate. DIF and IIF assays showed IgG/C3 deposition at the intercellular spaces (ICS) in most cases. A dual pattern with deposits at the basement membrane zone (BMZ) and ICS was described in one case (21). Autoantibodies against Dsc3 were found in 13 patients, while anti-Dsc1 antibodies were present in 7 patients. Anti-Dsc2 antibodies were only present in 3 patients who presented reactivity against the 3 desmocollin isoforms. Anti-Dsc3 antibodies were the only found in 10 patients and anti-Dsc1 were the only in 4. Autoantibodies against all 3 Dsc isoforms were detected in 3 patients who did not present a common phenotype or a more aggressive disease course (14, 18, 19). Autoantibodies against Dsc3 were detected in the 6 patients with mucosal involvement. There were no relevant comorbidities. Treatment and outcomes were reported in 13 cases which had mainly been treated with corticosteroids and dapsone. A partial to complete response was always observed. One patient was treated with rituximab 475 mg/m^2^ weekly for 4 weeks and another patient with 375 mg/m^2^ weekly for 4 weeks (2 courses) with good results (16, 21).

**Figure 1:**
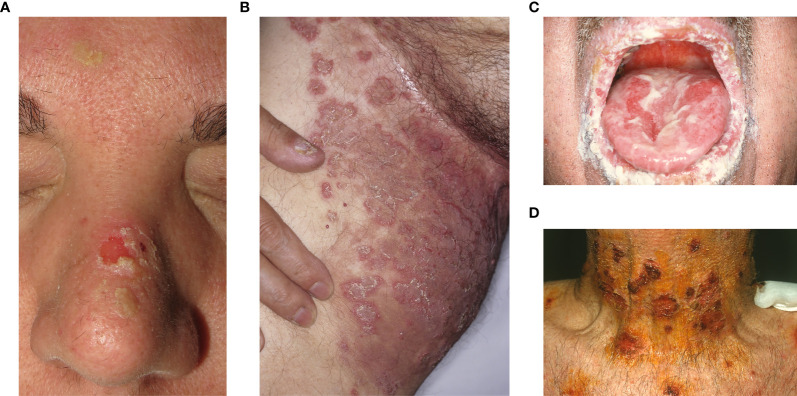
Clinical features. **(A)** A 33-year-old woman with exclusively anti-Dsc1 IgG antibodies and pemphigus foliaceous clinical features with erosions and crusts in the seborrheic areas. **(B)** A 66-year-old man with exclusively anti-Dsc1 IgA antibodies and annular plaques with pustules in the groin diagnosed as IgA pemphigus SPD-type. **(C, D)** A 70-year-old man with both anti-desmocollin 2,3, and anti-desmoglein 1,3 antibodies and mucocutaneous pemphigus vulgaris clinical features.

**Figure 2:**
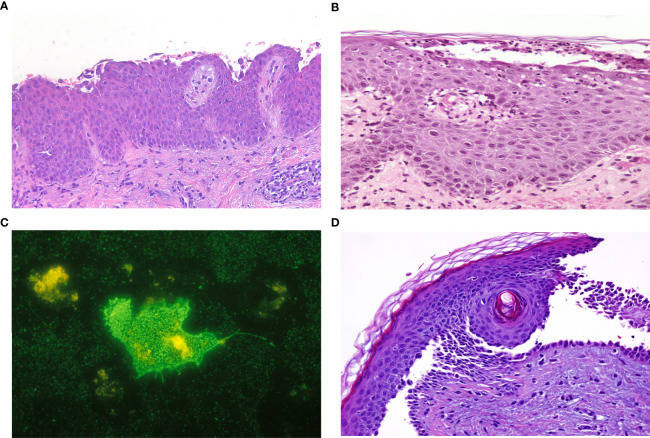
Histopathological features. **(A)** A 33-year-old woman with exclusively anti-Dsc1 IgG antibodies with complete detachment at the subcorneal layer, acantholytic cells and moderate lymphoplasmacytic perivascular dermal infiltrate (Hematoxylin–Eosin stain, x200). **(B, C)** A 66-year-old man with exclusively anti-Dsc1 IgA antibodies with a subcorneal neutrophilic pustule with dermal lymphocytic infiltrate (Hematoxylin–Eosin stain, x200) and a positive IgA indirect immunofluorescence with COS7 cells transfected with desmocollin 1 protein. **(D)** A 70-year-old man with both anti-desmocollin 2,3, and anti-desmoglein 1,3 antibodies and suprabasal acantholysis with dermal neutrophilic infiltrate (Hematoxylin–Eosin stain, x200).

A 33-year-old woman with exclusively anti-Dsc1 IgG antibodies and pemphigus foliaceous features is presented in **Figures 1A** and **2A**. First, treatment with prednisone and azathioprine, and after with prednisone and mycophenolate was administered. Currently, she is on complete remission off-therapy.

#### Patients With Exclusively IgA Anti-Desmocollin Autoantibodies

Wallach et al. in 1982 (22) described a new entity characterized by a vesiculopustular eruption with intraepidermal pustules and *in vivo* bound and/or circulating IgA antibodies. Since then, several similar cases have been reported under different denominations: IgA pemphigus, Intercellular IgA dermatosis and intercellular IgA vesiculopustular dermatosis. There are two main clinicopathological variants: SPD that presents as superficial pustules particularly on the intertriginous areas, subcorneal neutrophilic pustules, and IgA deposition in the upper epidermal ICS; and IEND presenting as sunflower-like inflammatory pustules, diffuse intraepidermal neutrophilic infiltrates, and IgA deposition involving all epidermal ICS. Hashimoto et al. (23) identified Dsc1 as the autoantigen of SPD-type, while the IEND-type target antigen has not yet been identified.

Between 2000 and 2021, 16 cases were described with exclusively IgA anti-Dsc autoantibodies (**Supplementary Table 2**) (23–36) Most patients were male (73%), with ages ranging from 9 to 94 years (median 64). SPD was the most common clinical pattern (10 patients). Mucosal involvement occurred in two patients, one with chronic lymphocytic leukemia with an overlap IEND-PNP (30) and another with PVeg features.‡ In both cases anti-Dsc2 was detected. Neutrophilic pustules/microabscesses were present in 10 cases and acantholysis in 9. When described, the infiltrate was composed of neutrophils and lymphocytes. DIF was negative in 1 case,‡ and a dual pattern was observed in the paraneoplastic case (30). Autoantibodies against Dsc1 were found in 12 of the 16 patients (8 with only these autoantibodies). Dsc2-3 autoantibodies were found less frequently (in 7 and 4 patients, respectively). Only 2 patients (one with initially weak reactivity against Dsc1) presented antibodies against the 3 Dsc isoforms (31, 32). Hematological diseases were detected in 4 patients without any predominance in anti-Dsc profile (28, 30, 33). Different therapies were administered, principally oral corticosteroids, dapsone and retinoids with mostly partial responses. The patient with chronic lymphocytic leukaemia presented a remission of both diseases with rituximab-fludarabine chemotherapy (30).

A 66-year-old man with a medical history of monoclonal IgA gammopathy and IgA pemphigus SPD-type with exclusively IgA Dsc1 autoantibodies is presented in **Figures 1B** and **2B, C**. Treatment with Dapsone, IVIG, tetracycline and isotretinoin was administered without response.

#### Patients With Exclusively IgG and IgA Anti-Desmocollin Autoantibodies

IgG/IgA pemphigus is a rare type of atypical pemphigus characterized by either *in vivo* and/or circulating IgG-IgA ICS antibodies. There is no consensus on whether IgG/IgA pemphigus is a subset of IgA pemphigus (37). In fact, in some of the reported IgA cases, IgG reactivity can also be appreciated in some tests such as DIF or IIF (**Supplementary Table 2**). Hashimoto et al. analysed 30 cases of this entity with distinctive clinical and immunopathological features which led them to consider it as a new disease, proposing the term Intercellular IgG/IgA dermatosis (IGAD). In this series underlying neoplasia occurred in 4 patients, autoimmune diseases in 5 and anti-Dsc antibodies were found in 19 (38).

There were only 5 patients with exclusively IgG and IgA anti-Dsc autoantibodies (**Supplementary Table 3**) (31, 38–40). Age range was between 39 and 80 (median 63) without sex predominance. PH was the most common clinical pattern and mucous membrane involvement was not reported. Pathological changes were variable; acantholysis was described in 3 cases and pustules in 4. DIF and IIF examination showed concomitant IgG/IgA deposition in 3 and 2 cases, respectively. No patients presented with all three isoforms of Dsc IgG/IgA autoantibodies. The reported treatments were systemic corticosteroids and dapsone with a certain degree of response.

### Patients With Anti-Desmocollin and Other Autoantibodies

In addition to anti-Dsc, other autoantibodies were detected in 55 reported cases. We have grouped them as:

#### Patients With Only Anti-Desmocollin and Anti-Desmoglein Autoantibodies

This profile was described in 35 patients (**Supplementary Table 4**) (8, 10, 38, 41–53). Age and sex were not always reported. Age ranged from 12 to 84 years (median 57). In patients where there is available information, skin involvement occurred in 31 of 34 cases, while mucous membranes were affected in 18 of 32 (3 exclusively). PV and PH were the most frequent phenotypes and pathological findings were reported in 26 cases. The most common features were acantholysis and intraepidermal eosinophilic/neutrophilic pustules. DIF was described in 23 cases, and was negative in one patient. IIF was detailed in 27 patients with negative results in 2. Autoantibodies against Dsg1, Dsg3 and Dsc1-3 were detected in a PH patient without DIF or treatment information (38). Treatment and outcome were described in 17 cases; dapsone and systemic corticosteroids were the most frequently employed, with variable responses. One patient received rituximab 375 mg/m^2^ weekly for 4 weeks (46) and another received rituximab 1 gram twice 2 weeks apart (3 courses) (38).

A 70-year-old man with mucocutaneous pemphigus vulgaris features and antibodies against desmocollin 2,3, and desmoglein 1,3 is presented in **Figures 1C, D** and **2D**. Systemic corticosteroids and azathioprine were started but the patient died from causes unrelated to his disease.

#### Patients With Anti-Desmocollin and Other Non-Desmoglein Autoantibodies

Dsc autoantibodies concomitantly with other non-desmoglein autoantibodies have been reported in 20 cases (**Supplementary Table 5**) (38, 47, 54–70). Overall, there were 12 females and 8 males with ages ranging between 11 and 83 years (median 66). Eight patients (40%) had a concurrent neoplasm: 4, lymphoproliferative diseases (55, 62, 63, 68), 3 had thymomas (66, 69, 70) and 1 presented with metastatic gastric cancer (57). Two thymoma-associated cases presented with PF (66, 70). Anti-plakin autoantibodies were detected in 12 patients, 7 with PNP features. Antibodies against different BMZ antigens were described in 18 cases. Treatments were varied and systemic corticosteroids were the most used. One patient received rituximab 1 gram twice 2 weeks apart with partial response (70). Six patients (30%) died during follow-up. Five of them had had an associated neoplasm, while the other had presented with PNP features and bronchiolitis obliterans without detectable malignancy.

## Discussion

In the present study of 93 anti-Dsc autoantibodies cases, the most common phenotype was PH in patients with either IgG or IgG/IgA anti-Dsc and anti-Dsc/Dsg autoantibodies; SPD in patients with IgA anti-Dsc autoantibodies; and PNP-like in those with antibodies against multiple antigens. A similar observation was described by Toosi et al. (71) when comparing clinicopathological findings of IgG/IgA, IgG and IgA anti-Dsg pemphigus since differences were appreciated between IgA and IgG/IgA but not between IgG and IgG/IgA.

Only 6% and 12% presented with PF and PV phenotype respectively, so we agree that the routine search for anti-Dsc antibodies should only be carried out in cases of atypical pemphigus as previously suggested (7, 72). Further, it remains unclear if anti-Dsc antibodies in PV patients could be as elevated as reported (44%) (6).

Thirteen of 17 patients with exclusive IgG anti-Dsc expressed Dsc3, and was the only antibody in 10. In contrast, anti-Dsc2 antibodies were only detected when reactivity against the three Dsc isoforms was detected. So it seems that Dsc3 would be the main antigen for this patient subtype. As described previously (23), Dsc1 autoantibodies were present in most cases of exclusive IgA anti-Dsc SPD-type. Most of patients with both IgG and IgA anti-Dsc antibodies had reactivity against the same isoform of desmocollin protein. Only one patient with IgG-IgA anti-Dsc3 antibodies also had only IgG against Dsc1 (38).

The presence of both anti-Dsc3 and anti-Dsg3 autoantibodies has been associated with a more severe PV phenotype and a slower response to prednisolone in a mouse model (73). In some cases anti-Dsc3 autoantibodies have been associated with a more severe epidermal detachment (74) or mucosal involvement (8, 50). The exact role of these anti-Dsc antibodies remains to be defined.

Among the patients with desmocollin together with other autoantibodies there were 12 patients with anti-plakin antibodies but only 7 were described as PNP. Ohzono et al. (75) described in a series of 104 cases of PNP that 11.5% showed no detectable neoplasia. They argued that this may be due to a limited sensitivity to detect tumors in early stages. Moreover, Sprecher suggested that non-neoplastic triggers may led to a clinical and immunological disorder identical to PNP (76). Another explanation could be that some of the anti-plakin antibodies detected were not pathogenic, so their presence could be due to a mechanism of epitope-spreading (77).

Mucosal involvement was present in 40 out of the 84 patients (48%) where information is available. Patients with exclusively IgG anti-Dsc autoantibodies and mucosal involvement always presented anti-Dsc3 autoantibodies, while the 2 patients with mucous manifestations and exclusive IgA anti-Dsc showed reactivity with Dsc2 without Dsc3. Currently, we have no explanation for these findings. Mucosal involvement was more frequent in patients where anti-Dsc were associated with other autoantibodies. This fact may be due to a synergistic action of the different autoantibodies or to a lower mucosal affinity of anti-Dsc antibodies.

Six cases (17, 20, 31, 52, 53, 64) developed a shift of clinical and/or autoantibodies pattern which could be regarded as an epitope-spreading phenomenon (77). Otherwise, the same antibody profile can be observed with different clinical patterns as seen in 3 patients with exclusive IgG anti-Dsc1-3 antibodies (PH, PF and PVeg). This fact suggests that autoantibodies may be produced independently (32) or target different epitopes.

Lymphoproliferative disorders and solid malignancies have been reported previously in 18% of patients with IgA pemphigus (78). In our review, a total of 17 (18%) neoplasms (mostly hematological) were described in all groups except in patients with exclusively Dsc IgG autoantibodies. Inflammatory bowel disease and other autoimmune diseases have been reported as associated with IgA pemphigus (78), but this association was infrequent in our study.

Regarding pathological findings (described in 82 cases), acantholysis was described in 44 patients and milder than in classical pemphigus. However, eosinophilic spongiosis and eosinophilic/neutrophilic intraepidermal pustules were more frequent. Dermal infiltrate is not a common finding in pemphigus, although lymphocytic infiltrate at the dermo-epidermal junction is a dominant feature in PNP (79). We consider that too little attention has been paid to dermal infiltrate in patients with anti-Dsc pemphigus, while in some articles it was not described but could be observed in the histological figures. The infiltrate was predominantly eosinophilic in IgG anti-Dsc cases, neutrophilic in IgA anti-Dsc cases and variable in patients with Dsc along with other autoantibodies. IgA autoantibodies may bind to the Fc receptor CD89 on monocytes and granulocytes, resulting in accumulation of neutrophils with a subsequent proteolytic cleavage of the keratinocyte cell-cell junction (80). Possibly by a similar mechanism, anti-Dsc autoantibodies could generate the clinicopathological pustules observed in these patients, as suggested by the concomitant change from PF or PV to PVeg with an eosinophilic/neutrophilic dermal infiltrate and the increased rate of anti-Dsc autoantibodies (20, 52). Rarely, anti-Dsc and anti-Dsg autoantibodies may be detected along with autoantibodies against different BMZ antigens. In these cases, histological findings of both acantholysis and subepidermal blistering may be simultaneously found in the same biopsy, together with a dual staining pattern by DIF examination.

DIF examination was negative in 5 of 74 reported cases (7%). Dual pattern was described in 14 patients, mostly in patients with autoantibodies against multiple antigens. Exclusively BMZ deposition occurred in only 2 patients. IIF was negative in 14 (18%) of 80 reported cases.

Treatment was described in 65 cases, with corticosteroids and dapsone as the most frequently administered. It is difficult to evaluate patient outcome due to the small number of patients, and the different clinical and serological patterns. Immunosuppressant agents were employed less often than in classic pemphigus. Globally, rituximab was administered to seven patients. Two patients (16, 21) with exclusively IgG Dsc autoantibodies showed a partial response with persistence of mucosal hypertrophy. One patient needed a second dose because of clinical relapse but after 3 follow-up years is in complete remission (21). The two patients with lymphoma and exclusively IgA anti-Dsc antibodies received rituximab-chemotherapy achieving total remission of both diseases (30, 33). Although rituximab could have a therapeutic role inhibiting autoantibody production, the total remission is more likely explained as the result of a paraneoplastic phenomenon because theoretically rituximab does not act against IgA autoreactive cells. Rituximab was also effective in a patient with refractory anti-Dsg/Dsc PV with a concomitant lingual squamous cell carcinoma (46) but failed to control either a severe case of IGAD in an adolescent (38) or a patient with a PF with concomitant thymoma (70).

## Conclusions

We should consider the presence of anti-Dsc antibodies in different circumstances: clinical features of PH, PVeg or atypical pemphigus; presence of pustules; classical pemphigus patients without detectable anti-Dsg antibodies; intraepithelial neutrophils/eosinophils or dermal neutrophilic/eosinophilic infiltrate on histological examination, and dual pattern DIF examination. When suspected, all available techniques should be employed. DIF positivity is the most reliable diagnostic test. Patients with exclusive IgG anti-Dsc autoantibodies, and those with both anti-Dsc and other non-Dsg autoantibodies, can be managed similarly to classical pemphigus and rituximab can be a good therapeutic option. In patients with IgA anti-Dsc antibodies systemic corticosteroids followed by dapsone or retinoids can be effective. Cases with IgA or IgG/IgA antibodies can be associated with malignancies (especially hematological) or autoimmune diseases.

## Author Contributions

XB-A, PI, and AE contributed to conception of the study, conducted the literature search, did the initial screen of articles and wrote the manuscript. All authors contributed to the article and approved the submitted version.

## Conflict of Interest

The authors declare that the research was conducted in the absence of any commercial or financial relationships that could be construed as a potential conflict of interest.

## Publisher’s Note

All claims expressed in this article are solely those of the authors and do not necessarily represent those of their affiliated organizations, or those of the publisher, the editors and the reviewers. Any product that may be evaluated in this article, or claim that may be made by its manufacturer, is not guaranteed or endorsed by the publisher.

## Abbreviations

AIBD, Autoimmune blistering diseases; BMZ, Basement membrane zone; Dsc, Desmocollin; Dsg, Desmoglein; DIF, Direct immunofluorescence; IIF, Indirect immunofluorescence; IGAD, Intercellular IgG/IgA dermatosis; ICS, Intercellular spaces; IEND, Intraepidermal neutrophilic dermatosis; PNP, Paraneoplastic pemphigus; PF, Pemphigus foliaceous; PH, Pemphigus herpetiformis; PV, Pemphigus vulgaris; PVeg, Pemphigus vegetans; SPD, Subcorneal pustular dermatosis.
