# Supplementary material for: Anti-Desmocollin Autoantibodies in Autoimmune Blistering Diseases
Source: Front Immunol. 2021 Sep 10;12:740820. doi: 10.3389/fimmu.2021.740820 (PMC8462461; doi:10.3389/fimmu.2021.740820)
Supplement: Supplementary file 1 [file Table_1.docx]

**Supplementary Table 1.** Patients with exclusively IgG desmocollin autoantibodies.

| **Author/year** | **Sex/Age** | **Clinic type** | **Skin** | **Mucous** | **Histopathology** | **DIF IC/BM** | **IIF** | **Dsc1** | **Dsc2** | **Dsc3** | **Treatment** | **Outcome** | **Others** |
| --- | --- | --- | --- | --- | --- | --- | --- | --- | --- | --- | --- | --- | --- |
| Mao/2010(8) | F/55 | MPV | No | Yes | Suprabasal acantholysis | NR | IgG | Neg | Neg | IgG | NR | NR | NR |
| Tateishi/2010(9) | M/83 | PH | Yes | NR | Intraepidermal pustules with Eo + Eosinophilic spongiosis | IgG/Neg | IgG | IgG | Neg | Neg | Dapsone, azathioprine, SC | CR | NR |
| Rafei/2011(10) | NR | PVeg | Yes | Yes | Acantholysis | IgG/Neg | IgG | Neg | Neg | IgG | NR | NR | NR |
| Rafei/2011(10) | NR | PH | Yes | Yes | Acantholysis | IgG/Neg | IgG | Neg | Neg | IgG | NR | NR | NR |
| Rafei/2011(10) | NR | PH | Yes | No | Acantholysis | IgG/Neg | IgG | Neg | Neg | IgG | NR | NR | NR |
| Hatano/2012(11) | F/79 | PH | Yes | No | Acantholysis + Eosinophilic spongiosis | IgG-C3/Neg | IgG | Neg | Neg | IgG | Nicotinamide, tetracycline, SC | CR | NR |
| Nakamura/2014(12) | M/57 | PH | Yes | No | Intraepidermal pustules with Eo + Eosinophilic spongiosis + Dermal infiltrate of Eo | IgG/Neg | IgG | Neg | Neg | IgG | Cyclosporine, SC | CR | No |
| Hong/2016(13) | F/76 | PH | Yes | No | Intraepidermal blister with Eo, Lym and Neu + Dermal infiltrate of Eo and Lym | IgG-C3/ Neg | IgG | Neg | Neg | IgG | Dapsone, SC | CR | No |
| Geller/2016(14) | F/11 | PF | Yes | No | Subcorneal blister + Acantholysis | IgG/Neg | Neg | IgG | IgG | IgG | SC | CR | NR |
| Ansai/2017(15) | F/63 | PH | Yes | No | Eosinophilic spongiosis + Dermal infiltrate of Eo | IgG-C3/ Neg | IgG | Neg | Neg | IgG | Dapsone | PR | Eosinophilia |
| Leventhal/2017(16) | F/17 | PVeg | No | Yes | Suprabasal acantholysis | IgG-C3/ Neg | IgG | Neg | Neg | IgG | Rituximab, surgery | PR | NR |
| Koga/2017(17) | M/70 | PH | Yes | No | Subcorneal pustules with Eo + Dermal infiltrate of Eo | IgG-C3/Neg | IgG | IgG | Neg | Neg | Dapsone, SC | CR | Eosinophilia, previous LABD |
| Tani/2018(18) | M/82 | PVeg | Yes | Yes | Intraepidermal pustules with Eo and Neu + Acantholysis + Dermal infiltrate of Eo, Lym and Neu | IgG/Neg | IgG | IgG | IgG | IgG | SC | CR | Eosinophilia |
| Tay/2018(19) | M/66 | PH | Yes | No | Intraepidermal vesicle with Eo + Eosinophilic spongiosis + Dermal infiltrate of Eo | IgG-C3/ Neg | IgG | IgG | IgG | IgG | Azathioprine, SC | CR | Uncontrolled DM |
| Yamaguchi/2018 (20) | M/39 | PVeg | Yes | No | Suprabasal bullae with Eo + Acantholysis + Dermal infiltrate of Eo | IgG-C3/ Neg | NR | IgG | Neg | Neg | SC, surgery | CR | Pemphigus vulgaris |
| Iranzo/2019(21) | F/70 | PVeg | Yes | Yes | Microabscesses with Neu and Eo + Suprabasal acantholysis + Dermal infiltrate of Eo and Lym | IgG-IgA/ IgG-IgA-C3 | Neg | Neg | Neg | IgG | Rituximab | PR | NR |
| New case reported in this manuscript as Figures 1 and 2 | F/33 | PF | Yes | No | Subcorneal acantholysis + Dermal infiltrate of Lym | IgG/Neg | IgG | IgG | Neg | Neg | SC, azathioprine, mycophenolate | CR | No |

*The authors original histopathology information has been completed with our review of the published images (if available).

*Abbreviations: BM, basement membrane; CR, complete response; DIF, direct immunofluorescence; DM, diabetes mellitus; Dsc, desmocollin; Eo, eosinophils; F, female; IC, intercellular; IIF, indirect immunofluorescence; LABD, linear IgA bullous disease; Lym, lymphocyte; M, male; MPV, mucosal pemphigus vulgaris; Neg, negative; Neu, neutrophils; NR, no reported; PF, pemphigus foliaceus; PH, pemphigus herpetiformis; PR, partial response; PVeg, pemphigus vegetans; SC, systemic corticoids.*
