# Supplementary material for: Anti-Desmocollin Autoantibodies in Autoimmune Blistering Diseases
Source: Front Immunol. 2021 Sep 10;12:740820. doi: 10.3389/fimmu.2021.740820 (PMC8462461; doi:10.3389/fimmu.2021.740820)
Supplement: Supplementary file 2 [file Table_2.docx]

**Supplementary Table 2.** Patients with exclusively IgA desmocollin autoantibodies.

| **Author/year** | **Sex/Age** | **Clinic Type** | **Skin** | **Mucous** | **Histopathology** | **DIF IC/BM** | **IIF** | **Dsc1** | **Dsc2** | **Dsc3** | **Treatment** | **Outcome** | **Others** |
| --- | --- | --- | --- | --- | --- | --- | --- | --- | --- | --- | --- | --- | --- |
| Yasuda/2000(24) | M/40 | SPD | Yes | No | Subcorneal pustule with Neu + Acantholysis + Dermal infiltrate of Lym and Neu | IgA/Neg | IgA | IgA | Neg | Neg | Dapsone, SC, etretinate | PR | NR |
| Gruss/2000(25) | M/65 | SPD | Yes | No | Subcorneal pustule with Neu + Acantholysis | IgA/Neg | Neg | IgA | Neg | Neg | Isotretinoin | PR | NR |
| Botella/2001(26) | F/74 | SPD | Yes | No | Subcorneal pustule with Neu + Dermal infiltrate of Lym and Neu | Neg/Neg | IgA | IgA | Neg | Neg | Dapsone, acitretin | PR | No |
| Hashimoto/2002(27) | M/63 | SPD | Yes | No | Subcorneal pustule with Neu | IgA/Neg | IgA | IgA | Neg | Neg | SC, etretinate | PR | NR |
| Ruiz-Genao/2002(28) | M/67 | SPD | Yes | No | Subcorneal pustule + Acantholysis | IgA/Neg | Neg | IgA | Neg | Neg | Dapsone, colchicine, acitretin, melphalan | CR | IgA monoclonal gammopathy |
| De Oliveira/2003(29) | F/29 | SPD | Yes | No | Subcorneal pustule + Acantholysis | IgA/Neg | Neg | IgA | Neg | Neg | Dapsone, SC, colchicine | PR | No |
| De Oliveira/2003(29) | M/9 | PH | Yes | No | Subcorneal vesicle with Neu + Acantholysis | IgA/Neg | Neg | IgA | Neg | Neg | Dapsone, colchicine | PR | No |
| Taintor/2007(30) | M/79 | IEND-PNP | Yes | Yes | Intraepidermal vesicle with Neu + Acantholysis + Dermal infiltrate of Lym and Neu | IgA/IgG | IgA | Neg | IgA | Neg | Rituximab, fludarabine | CR | Chronic lymphocytic leukemia |
| Müller/2009(31) | M/80 | PH | Yes | NR | Eosinophilic spongiosis | C3/Neg | NR | IgA | IgA | IgA | NR | NR | NR |
| Düker/2009(32) | F/94 | SPD | Yes | No | Subcorneal pustule with Neu + Acantholysis + Dermal infiltrate of Neu | IgA/Neg | IgA | IgA | IgA | IgA | Dapsone | CR | NR |
| Asahina/2013(33) | M/69 | SPD | Yes | No | Subcorneal and mid-epidermis pustule with Neu + Acantholysis + Dermal infiltrate of Neu | IgA/Neg | IgA | Neg | IgA | IgA | Rituximab-CHOP | CR | Lymphoma |
| Arai/2013(34) | M/56 | PH | Yes | No | Eosinophilic spongiosis + Dermal infiltrate of Eo, Lym and Neu | IgA/Neg | IgA | IgA | IgA | Neg | Nicotinic acid, minocycline, SC | CR | Eosinophilia |
| Kiritsi/2017(35) | F/50 | SPD | Yes | No | Subcorneal vesicle with Neu + Acantholysis | IgA/Neg | NR | Neg | Neg | IgA | Colchicine | CR | NR |
| Hashimoto/2018(38) | M/56 | IGAD | Yes | No | Spongiosis | IgA/Neg | IgA-IgG | Neg | IgA | Neg | Dapsone, SC | CR | No |
| Bosch/2021(36‡) | M/56 | PVeg | No | Yes | Spongiosis with neutrophilic microabscesses | Neg/Neg | IgA | IgA | IgA | Neg | IVIG | CR | Uveitic macular edema |
| New case reported in this manuscript as Figures 1 and 2 | M/66 | SPD | Yes | No | Subcorneal pustule with Neu + Dermal infiltrate of Lym | IgA/Neg | Neg | IgA | Neg | Neg | Dapsone, IVIG, tetracycline, isotretinoin | No response | Endothoracic goiter, monoclonal IgA gammopathy |

^‡^Article accepted in press at Annals of Dermatology (ISSN 1013-9087).

*The authors original histopathology information has been completed with our review of the published images (if available).

*Abbreviations: BM, basement membrane; CHOP, cyclophosphamide-doxorubicin-vincristine-prednisolone; CR, complete response; DIF, direct immunofluorescence; Dsc, desmocollin; Eo, eosinophils; F, female; IC, intercellular; IEND, intraepidermal neutrophilic dermatosis; IGAD, intercellular IgG/IgA dermatosis; IIF, indirect immunofluorescence; IVIG, intravenous immunoglobulins; Lym, lymphocytes; M, male; Neg, negative; Neu, neutrophils; NR, no reported; PH, pemphigus herpetiformis; PNP, paraneoplastic pemphigus; PR, partial response; PVeg, pemphigus vegetans; SC, systemic corticoids; SPD, subcorneal pustular dermatosis.*
