# Supplementary material for: Anti-Desmocollin Autoantibodies in Autoimmune Blistering Diseases
Source: Front Immunol. 2021 Sep 10;12:740820. doi: 10.3389/fimmu.2021.740820 (PMC8462461; doi:10.3389/fimmu.2021.740820)
Supplement: Supplementary file 3 [file Table_3.docx]

**Supplementary Table 3.** Patients with exclusively IgG and IgA desmocollin autoantibodies.

| **Author/year** | **Sex/Age** | **Clinic Type** | **Skin** | **Mucous** | **Histopathology** | **DIF IC/BM** | **IIF** | **Dsc1** | **Dsc2** | **Dsc3** | **Treatment** | **Outcome** | **Others** |
| --- | --- | --- | --- | --- | --- | --- | --- | --- | --- | --- | --- | --- | --- |
| Chorzelski/1994 (39) | M/63 | PH | Yes | No | Subcorneal pustule with Neu + Acantholysis | IgA-IgG/ Neg | IgA-IgG | IgA-IgG | IgA-IgG | Neg | Dapsone | CR | Lung cancer |
| Heng/2006(40) | F/39 | IEND | Yes | NR | Intraepidermal pustule with Neu + Acantholysis | IgA-IgG/ Neg | IgA-IgG | IgA-IgG | Neg | Neg | Dapsone | PR | NR |
| Müller/2009(31) | NR | Atypical pemphigus | Yes | NR | NR | NR | NR | IgA-IgG | Neg | Neg | NR | NR | NR |
| Hashimoto/2018 (38) | F/80 | PH | Yes | NR | Subcorneal pustule with Eo + Spongiosis Eo | IgG-C3/ Neg | Neg | IgG | Neg | IgA-IgG | SC | CR | Sjogren |
| Hashimoto/2018 (38) | M/64 | Erythroderma, blisters and pustules | Yes | NR | Intraepidermal pustule with Neu + Acantholysis | IgA-IgG-C3 /Neg | Neg | Neg | Neg | IgA-IgG | NR | NR | Lymphoma |

*The authors original histopathology information has been completed with our review of the published images (if available).

*Abbreviations: CR, complete response; DIF, direct immunofluorescence; Dsc, desmocollin; Eo, eosinophils; F, female; IC, intercellular; IEN, intraepidermal neutrophilic dermatosis; IIF, indirect immunofluorescence; M, male; Neg, negative; Neu, neutrophils; NR, no reported; PH, pemphigus herpetiformis; PR, partial response; SC, systemic corticoids.*
