# Supplementary material for: Anti-Desmocollin Autoantibodies in Autoimmune Blistering Diseases
Source: Front Immunol. 2021 Sep 10;12:740820. doi: 10.3389/fimmu.2021.740820 (PMC8462461; doi:10.3389/fimmu.2021.740820)
Supplement: Supplementary file 4 [file Table_4.docx]

**Supplementary Table 4.** Patients with only desmocollin and desmoglein autoantibodies.

| **Author/year** | **Sex/Age** | **Clinic Type** | **Skin** | **Mucous** | **Histopathology** | **DIF IC/ BM** | **IIF** | **Dsg1** | **Dsg3** | **Dsc1** | **Dsc2** | **Dsc3** | **Treatment** | **Outcome** | **Others** |
| --- | --- | --- | --- | --- | --- | --- | --- | --- | --- | --- | --- | --- | --- | --- | --- |
| Hashimoto/1994(41) | F/67 | PVeg | Yes | Yes | Intraepidermal pustules with Eo | IgG/Neg | IgG | IgG | IgG | IgG | IgG | NR | NR | NR | NR |
| Hashimoto/1994(41) | F/33 | PVeg | Yes | Yes | Suprabasal and intraepidermal blister with Eo + Acantholysis | IgG/Neg | IgG | IgG | IgG | IgG | IgG | NR | NR | NR | NR |
| Hashimoto/1995(42) | M/38 | PV | Yes | Yes | Suprabasal blister + Acantholysis | IgG-C3/ Neg | IgG | IgG, IgA | IgG | Neg | IgG | Neg | SC, cyclosporine | CR | NR |
| Kozlowska/2003(43) | M/42 | PH | Yes | No | Intraepidermal blister with Eo and Neu + Eosinophilic spongiosis + Acantholysis | IgA-IgG/ Neg | IgA-IgG | IgG-IgA | Neg | IgG | Neg | IgG | Hydroxychloroquine, nicotinamide, SC, dapsone | PR | Lupus anticoagulant, anticardiolipin antibody |
| Kopp/2006(44) | M/48 | Generalized bullae-pustules | Yes | No | Subcorneal pustule + Dermal infiltrate of Eo and Lym | IgA/Neg | Neg | IgA | Neg | IgA | Neg | Neg | Dapsone | CR | No |
| Mao/2010(8) | NR | PV | Yes | Yes | NR | NR | NR | Neg | IgG | Neg | Neg | IgG | NR | NR | NR |
| Mao/2010(8) | NR | PV | No | Yes | NR | NR | NR | Neg | IgG | Neg | Neg | IgG | NR | NR | NR |
| Mao/2010(8) | NR | PV | Yes | Yes | NR | NR | NR | IgG | IgG | Neg | Neg | IgG | NR | NR | NR |
| Mao/2010(8) | NR | PV | Yes | Yes | NR | NR | NR | IgG | IgG | Neg | Neg | IgG | NR | NR | NR |
| Mao/2010(8) | NR | PV | Yes | Yes | NR | NR | NR | IgG | IgG | Neg | Neg | IgG | NR | NR | NR |
| Rafei/2011(10) | NR | PVeg | Yes | No | Acantholysis | IgG/Neg | IgG | IgG | Neg | Neg | Neg | IgG | NR | NR | NR |
| Hosoda/2012(45) | F/62 | PF/PH | Yes | No | Neutrophilic spongiosis | IgG-IgA/ Neg | IgG-IgA | IgG-IgA | Neg | IgG | IgG | IgG | SC, dapsone | PR | No |
| Kim/2013(46) | M/54 | PV | Yes | Yes | NR | NR | NR | Neg | IgG | IgG | IgG | IgG | SC, azathioprine, Rituximab, IVIG, cyclophosphamide | CR | Lingual SCC |
| Saruta/2013(47) | F/80 | PVeg | Yes | No | Intraepidermal pustules with Eo and Neu + Eosinophilic spongiosis + Acantholysis + Dermal infiltrate of Eo and Neu | C3/Neg | IgG | IgG | Neg | Neg | Neg | IgG | SC | CR | No |
| Matsukura/2014(48) | F/84 | PH | Yes | No | Intraepidermal pustules with Eo and Neu + Spongiosis + Acantholysis + Dermal infiltrate | IgG-C3/ Neg | IgG | IgG | Neg | Neg | Neg | IgG | SC, dapsone, IVIG, plasmapheresis | CR | Eosinophilia, elevated IgE |
| On/2015(49) | M/51 | PH | Yes | No | Intraepidermal pustules with Eo and Neu + Acantholysis + Dermal infiltrate with Eo and Lym | IgG-C3/ Neg | IgG | IgG | Neg | IgG | Neg | Neg | SC, dapsone | PR | No |
| Makino/2015(50) | F/49 | PV | No | Yes | Acantholysis + Dermal infiltrate with Lym | IgG/Neg | IgG | Neg | IgG | Neg | IgG | IgG | SC, IVIG | PR | No |
| Kamiya/2016(51) | M/56 | PV | No | Yes | Acantholysis + Dermal infiltrate | IgG/Neg | NR | Neg | IgG-IgA | Neg | Neg | IgG | SC, IVIG | CR | No |
| Matsuyama/2018(52) | M/59 | PVeg | Yes | No | Acantholysis + Eosinophilic spongiosis + Dermal infiltrate of Eo and Neu | IgG/Neg | NR | IgG | Neg | IgG | IgG | IgG | SC, minocycline | CR | Eosinophilia, PF |
| Hashimoto/2018(38) | F/39 | PH | Yes | NR | NR | NR | IgG-IgA | IgG-IgA | Neg | IgG-IgA | Neg | IgG | NR | NR | Uterine neoplasm |
| Hashimoto/2018(38) | M/NR | PV | Yes | Yes | Intraepidermal pustules with Neu | IgG-IgA-C3/Neg | IgG-IgA | IgG-IgA | Neg | Neg | IgA | Neg | NR | NR | NR |
| Hashimoto/2018(38) | M/71 | Eritrodermia + LP-like oral lesions | Yes | Yes | Intraepidermal blister | NR | IgG-IgA | IgG-IgA | Neg | IgA | Neg | Neg | NR | NR | Myasthenia Gravis |
| Hashimoto/2018(38) | F/49 | PH | Yes | NR | NR | NR | IgG-IgA | IgG-IgA | IgG-IgA | IgG-IgA | IgG-IgA | IgG-IgA | NR | NR | No |
| Hashimoto/2018(38) | F/75 | PF/PH | Yes | No | Intraepidermal blister | NR | IgG-IgA | IgG-IgA | Neg | IgA | IgG | IgG | NR | NR | NR |
| Hashimoto/2018(38) | F/61 | LAD | Yes | No | Intraepidermal pustules with Neu | Neg/Neg | IgG-IgA | Neg | IgA | Neg | IgG | Neg | NR | NR | NR |
| Hashimoto/2018(38) | M/NR | LAD | Yes | Yes | Intraepidermal pustules with Neu | NR | IgG-IgA | IgG-IgA | IgG-IgA | Neg | IgA | IgA | NR | NR | Sweet Sd |
| Hashimoto/2018(38) | NR | LAD | NR | NR | NR | NR | IgG-IgA | IgG-IgA | Neg | Neg | Neg | IgG | NR | NR | Harada disease |
| Hashimoto/2018(38) | F/58 | PH | Yes | Yes | Acantholysis + Neutrophilic spongiosis + Dermal infiltrate of Lym | IgG-IgA/ Neg | IgG | IgG-IgA | IgG-IgA | Neg | IgA | Neg | SC, dapsone | CR | Breast cancer |
| Hashimoto/2018(38) | F/66 | IGAD | Yes | Yes | Intraepidermal blister with Eo and Neu | IgG-IgA/ Neg | IgG-IgA | IgG-IgA | IgG-IgA | Neg | IgG | Neg | NR | NR | NR |
| Hashimoto/2018(38) | M/84 | IGAD | Yes | No | Subcorneal pustule with Eo and Neu + Dermal infiltrate of Eo and Neu | IgG-IgA/ Neg | Neg | Neg | IgG | Neg | Neg | IgG-IgA | NR | NR | Pneumoconiosis |
| Hashimoto/2018(38) | F/39 | PH | Yes | No | Intraepidermal pustules with Eo | IgG-IgA/ C3 | IgG-IgA | IgG-IgA | Neg | IgG | Neg | Neg | SC | CR | Rheumatoid arthritis |
| Hashimoto/2018(38) | M/65 | IGAD | Yes | No | Subcorneal vesicle + Acantholysis | IgG-IgA/ Neg | IgG-IgA | IgG-IgA | Neg | Neg | Neg | IgG | SC, dapsone | CR | NR |
| Hashimoto/2018(38) | M/12 | IGAD | Yes | No | Subcorneal pustules with Neu + Acantholysis | IgG-IgA-C3/C3 | IgA | Neg | IgG-IgA | Neg | IgG-IgA | IgG-IgA | SC, dapsone, mycophenolate, Rituximab, isotretinoin | PR | NR |
| Shimokata/2020(53) | M/51 | Atypical pemphigus | Yes | Yes | Suprabasal blister with Eo, Lym and Neu + Acantholysis | IgG-C3/ Neg | IgG | Neg | IgG | Neg | IgG | IgG | SC, IVIG, azathioprine | PR | NR |
| New case reported in this manuscript as Figures 1 and 2 | M/70 | PV | Yes | Yes | Acantholysis+ Dermal infiltrate of Neu | IgG-C3/ Neg | IgG | IgG | IgG | Neg | IgG | IgG | SC, azathioprine | Death | NR |

*The authors original histopathology information has been completed with our review of the published images (if available).

*Abbreviations: CR, complete response; DIF, direct immunofluorescence; Dsc, desmocollin; Dsg, desmoglein; Eo, eosinophils; F, female; IC, intercellular; IGAD, intercellular IgG/IgA dermatosis; IIF, indirect immunofluorescence; IVIG, intravenous immunoglobulins; LAD, linear IgA dermatosis; LP, lichen planus; Lym, lymphocytes; M, male; Neg, negative; Neu, neutrophils; NR, no reported; PF, pemphigus foliaceus; PH, pemphigus herpetiformis; PR, partial response; PV, pemphigus vulgaris; PVeg, Pemphigus vegetans; SC, systemic corticoids.*
