# Supplementary material for: Anti-Desmocollin Autoantibodies in Autoimmune Blistering Diseases
Source: Front Immunol. 2021 Sep 10;12:740820. doi: 10.3389/fimmu.2021.740820 (PMC8462461; doi:10.3389/fimmu.2021.740820)
Supplement: Supplementary file 5 [file Table_5.docx]

**Supplementary Table 5.** Patients with desmocollin and other autoantibodies.

| **Author/year** | **Sex/Age** | **Clinic Type** | **Skin** | **Mucous** | **Histopathology** | **DIF IC/BM** | **IIF IC/BM** | **Rat Bladder** | **IB/IP** | **ELISA** | **cDNA transfection** | **Treatment** | **Outcome** | **Others** |
| --- | --- | --- | --- | --- | --- | --- | --- | --- | --- | --- | --- | --- | --- | --- |
| Gooptu/1999(54) | F/11 | PNP | Yes | Yes | Subepidermal pustules + epidermal microabscesses + Dermal infiltrate | IgG-IgA/ IgG-IgA-C3 | IgG-IgA/ Neg | NR | Desmoplakin I-II, Dsc | Dsg3 | NR | Dapsone, sulphapyridine, SC, colchicine, IVIG, azathioprine | PR | NR |
| Preisz/2004(55) | F/48 | PNP | Yes | Yes | Interface dermatitis + Acantholysis | IgG-IgA-C3/ C3 | IgG/Neg | IgG-IgA | Envoplakin, periplakin, BP180 | Dsg3, Dsc3 | Dsc2, Dsc3 | SC, IVIG, cyclophosphamide | Death | Follicular Lymphoma |
| Bolling/2007(56) | M/63 | BP | Yes | No | Suprabasal blister + Acantholysis + Eosinophilic spongiosis + Dermal infiltrate of Eo and Lym | IgG/IgG-C3 | IgG/ IgG-IgA | NR | BP230, LAD-1, Dsc3 | BP180 | NR | Tetracycline, Nicotinamide | CR | Keratoderma |
| Endo/2010(57) | M/53 | PH | Yes | No | Intraepidermal pustules with Eo and Neu | Neg/Neg | IgG/Neg | IgG | Dsc, LAD-1, desmoplakin | Neg | Dsc1-3 | SC | Death | Metastatic gastric cancer |
| Ueda/2013(58) | M/68 | PH | Yes | Yes | Subepidermal blister with Eo and Neu + Epidermal blister with Eo + Eosinophilic spongiosis | IgG-C3/ IgG-C3 | IgG-IgA/ IgG | NR | BP230, LAD-1 | Dsc1, Dsc3 | Dsc1, Dsc3 | Dapsone, SC | CR | ANA+ |
| Ueda/2013(59) | F/79 | PNP | Yes | Yes | Epidermal necrosis + Dermal infiltrate of Eo and Neu | NR | IgA/ IgG-IgA | NR | BP230, periplakin | Dsg1, Dsg3 | Dsc3 | SC, IVIG, plasmapheresis | Death | No |
| Ohata/2013(60) | M/63 | PH | Yes | No | Intraepidermal and subepidermal blister with Eo + Eosinophilic spongiosis + Dermal infiltrate of Eo | IgG-IgA-C3/ C3 | IgG/ IgA-IgA | IgG | Periplakin, BP180 C-Terminal, Lam-332 | Dsc1 | NR | SC | CR | Eosinophilia |
| Ohata/2013(61) | F/83 | PH-BP | Yes | Yes | Intraepidermal and subepidermal vesicles with Eo + Eosinophilic spongiosis + Dermal infiltrate of Eo | IgG/C3 | IgG/Neg | NR | BP180 | BP180, Dsg1, Dsg3, Dsc2-3 | Dsc1-3 | SC | CR | Eosinophilia |
| Saruta/2013(47) | F/70 | PVeg | Yes | Yes | Eosinophilic pustules with Eo and Neu | Neg/Neg | IgG/Neg | NR | Dsc, BP230, Periplakin | Dsc2-3 | Dsc3 | SC | CR | Eosinophilia, ANA |
| Gallo/2014(62) | F/81 | PNP | Yes | Yes | Eosinophilic spongiosis + Acantholysis | IgG-C3/Neg | IgG/Neg | NR | Envoplakin, periplakin | Dsg3, Dsc2-3 | NR | Azathioprine, SC | Death | Follicular lymphoma |
| Yashiro/2014(63) | M/69 | PNP | Yes | Yes | Subepidermal blister + Dermal infiltrate of Lym | IgG-IgA/IgA | IgA/ IgG-IgA | IgG-IgA | BP230, Lam-332, Col VII, Periplakin Envoplakin | Dsc1 | NR | SC, chemotherapy | CR | Angioimmunoblastic T-Cell Lymphoma |
| Uchiyama/2014(64) | F/78 | Atypical PV | Yes | Yes | Intraepidermal vesicle with Eo and Neu + Acantholysis | IgG-IgA-C3/ C3 | Neg/Neg | NR | Neg | BP180, Dsc1-3 | Dsc1 | SC | CR | Ulcerous colitis, ANA |
| Geller/2014(65) | F/74 | PH | Yes | No | Subcorneal pustule with Neu, Eo + Subepidermal blister with Neu + Acantholysis + Dermal infiltrate | Neg/C3 | IgA/ IgG-IgA | NR | Dsc2-3, BP180 | Neg | NR | Dapsone | CR | NR |
| Tsuchisaka/2015(66) | F/82 | PF | Yes | No | Acantholysis at upper epidermis | NR | NR | NR | Dsg2-3, Dsc2-3, periplakin, Lam- γ1 | Dsg1 | NR | NR | Death | Thymoma |
| Inoue/2016(67) | M/50 | PVeg | Yes | Yes | Epidermal pustule with Eo + Eosinophilic spongiosis + Dermal infiltrate of Eo | NR | Neg/Neg | NR | Dsg3, Periplakin | Dsg3, Dsc3, BP180 | NR | Indomethacin, minocycline | CR | Eisenmenger disease, Eosinophilia |
| Otsuka/2016(68) | M/63 | PNP | Yes | Yes | Interface dermatitis + Dermal infiltrate of Lym | IgG-C3/C3 | IgA/Neg | IgG-IgA | Envoplakin, Periplakin, Dsg, BP180, BP230 | Dsg1, Dsg3, Dsc3 | NR | SC, IVIG | Death | Non-Hodgkin Lymphoma |
| Lim/2017(69) | F/56 | PNP | Yes | Yes | Interface dermatitis | NR | IgG/Neg | IgG | Envoplakin, periplakin, Lam-γ1 | Dsg1, Dsc1-3 | NR | SC, cyclosporine, mycophenolate mofetil, surgery + radiotherapy | PR | Thymoma |
| Hashimoto/2018(38) | F/79 | MMP-PNP | Yes | Yes | Epidermal blister with Neu | NR | IgA/ IgG-IgA | NR | BP180, BP230 | Dsg1, Dsg3, Dsc1-3 | Dsc1 | NR | NR | NR |
| Hashimoto/2018(38) | M/11 | IGAD | Yes | Yes | NR | Neg/ IgG-IgA | IgG-IgA/ IgG-IgA | IgG-IgA | LAD-1 | Dsg1, Dsg3, Dsc1-3 | Neg | NR | NR | NR |
| Solimani/2019(70) | F/51 | PF-GVHD | Yes | No | Spongiosis with neutrophilic microabscesses | IgG-C3/ IgG-C3 | Neg/Neg | Neg | Lam332 | Dsg1, Dsc1 | Neg | IVIG, Rituximab | PR | Thymoma , M Gravis |

*The authors original histopathology information has been completed with our review of the published images (if available).

*Abbreviations: AP, annular plaques; BM, basement membrane; CR, complete response; DIF, direct immunofluorescence; Dsc, desmocollin; Dsg, desmoglein; Eo, eosinophils; F, Female; GVHD, graft versus host disease; IB, immunoblotting; IC, intercellular; IGAD, intercellular IgG/IgA dermatosis; IIF, Indirect immunofluorescence; IP, immunoprecipitation; IVIG, intravenous immunoglobulins; Lam, laminin; LP, lichen planus; Lym, lymphocytes; M, Male; Neu, neutrophils; MMP, mucous membrane pemphigoid; NR, No reported; PF, pemphigus foliaceus; PH, Pemphigus herpetiformis; PNP, paraneoplastic pemphigus; PR, partial response; PVeg, Pemphigus vegetans; SC, systemic corticoids.*
